# Supplementary material for: Profiling DNA methylation differences between inbred mouse strains on the Illumina Human Infinium MethylationEPIC microarray
Source: PLoS One. 2018 Mar 12;13(3):e0193496. doi: 10.1371/journal.pone.0193496 (PMC5846735; doi:10.1371/journal.pone.0193496)
Supplement: S1 Fig — (A) Hierarchical clustering of β-values for the 13665 conserve probes on the Illumina Infinium MethylationEPIC shows a clear separation between the mouse and human samples. For the mouse samples, DBA/2J and C57BL/6J samples group separately. Mouse sample 1 (M1) is an outlier and has low average signal intensity compared to the other mouse samples. (B) Principal component analysis was performed for the 11 mouse samples using β-values for the 13665 conserve probes. A scatter plot of the first two principal components, PC1 and PC2, clearly demonstrate the outlier status of mouse sample 1 (arrow) and this sample was excluded in the differential methylation analyses. (DOCX) [file pone.0193496.s002.docx]

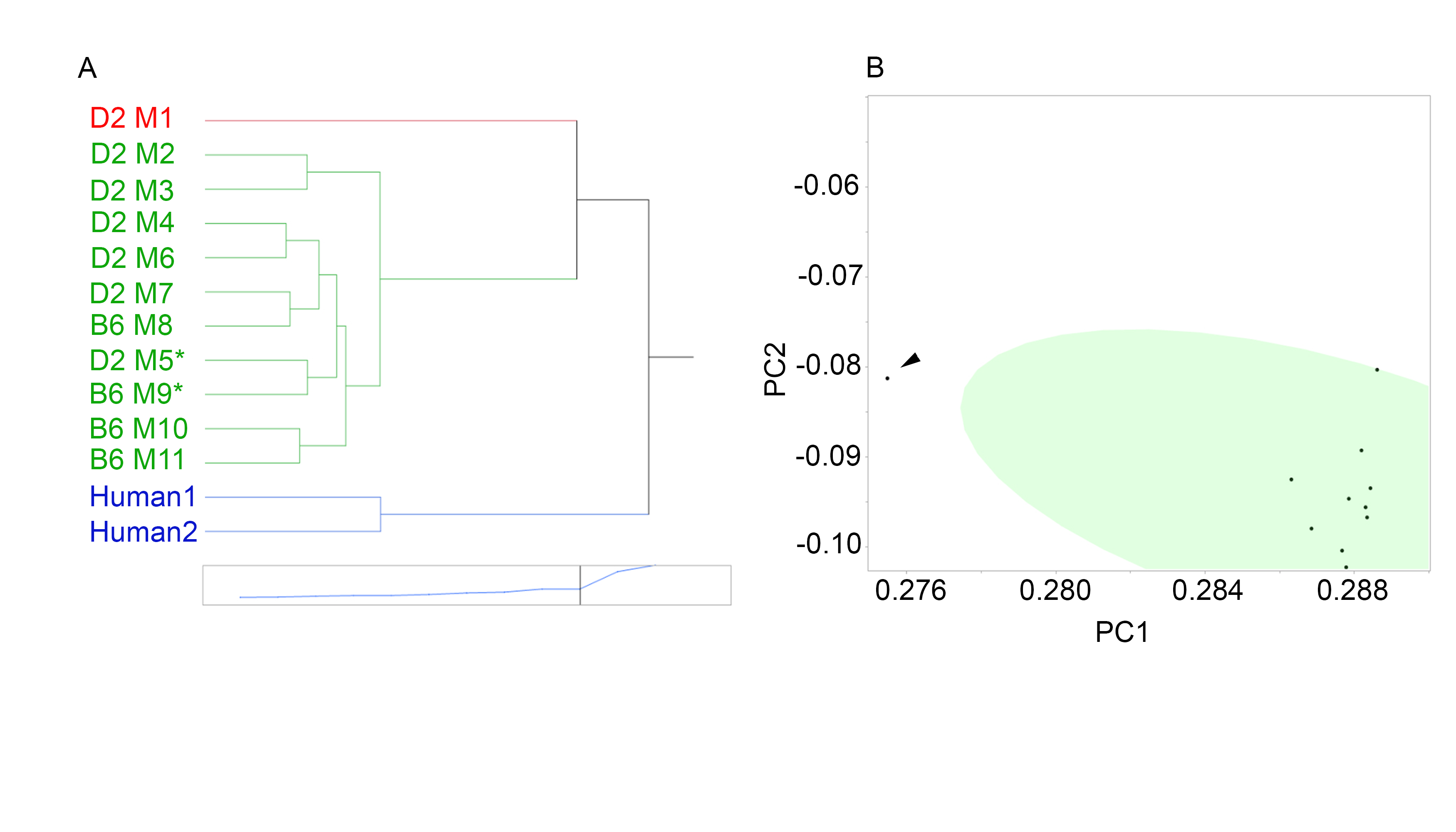


**S1 Fig. Quality check for samples using β-values.**

**(A)** Hierarchical clustering of β-values for the 13665 conserve probes on the Illumina Infinium MethylationEPIC shows a clear separation between the mouse and human samples. For the mouse samples, DBA/2J and C57BL/6J samples group separately. Mouse sample 1 (M1) is an outlier and has low average signal intensity compared to the other mouse samples. **(B)** Principal component analysis was performed for the 11 mouse samples using β-values for the 13665 conserve probes. A scatter plot of the first two principal components, PC1 and PC2, clearly demonstrate the outlier status of mouse sample 1 (arrow) and this sample was excluded in the differential methylation analyses.
